# Differentiation of Traumatic Osteoporotic and Non-Osteoporotic Vertebral AO A3 Fractures by Analyzing the Posterior Edge Morphology—A Retrospective Feasibility Study

**DOI:** 10.3390/jcm9123910

**Published:** 2020-12-02

**Authors:** Thomas Vordemvenne, Dirk Wähnert, Sebastian Klingebiel, Jens Lohmaier, René Hartensuer, Michael J. Raschke, Steffen Roßlenbroich

**Affiliations:** 1 Department of Trauma and Orthopedic Surgery, Protestant Hospital of Bethel Foundation, University Hospital OWL of Bielefeld University, Campus Bielefeld-Bethel, Burgsteig 13, 33617 Bielefeld, Germany; dirk.waehnert@evkb.de (D.W.); jens.lohmaier@evkb.de (J.L.); 2 Department of Orthopedics, University Hospital Muenster, Albert-Schweitzer-Campus 1, 48149 Muenster, Germany; sebastian.klingebiel@ukmuenster.de; 3 Department of Trauma, Hand and Reconstructive Surgery, University Hospital Muenster, Albert-Schweitzer-Campus 1, Building W1, 48149 Muenster, Germany; rene.hartensuer@ukmuenster.de (R.H.); michael.raschke@ukmuenster.de (M.J.R.); steffen.rosslenbroich@ukmuenster.de (S.R.)

**Keywords:** traumatic vertebral fracture, osteoporosis, posterior edge, morphology, Hounsfield units

## Abstract

Background: Differentiation between traumatic osteoporotic and non-osteoporotic vertebral fractures is crucial for optimal therapy planning. We postulated that the morphology of the posterior edge of the cranial fragment of A3 vertebral fractures is different in these entities. Therefore, the purpose of this study is to develop and validate a simple method to differentiate between osteoporotic and non-osteoporotic A3 vertebral fractures by morphological analysis. Methods: A total of 86 computer tomography scans of AO Type A3 (cranial burst) vertebral body fractures (52 non-osteoporotic, 34 osteoporotic) were included in this retrospective study. Posterior edge morphology was analyzed using the sagittal paramedian slice with the most prominent shaped bulging. Later, the degree of bulging of the posterior edge fragment was quantified using a geometric approach. Additionally, the Hounsfield units of the broken vertebral body, the vertebra above, and the vertebra below the fracture were measured. Results: We found significant differences in the extent of bulging comparing osteoporotic and non-osteoporotic fractures in our cohort. Using the presented method, sensitivity was 100%, specificity was 96%. The positive predictive value (PPV) was 94%. In contrast, by evaluating the Hounsfield units, sensitivity was 94%, specificity 94% and the PPV was 91%. Conclusions: Our method of analysis of the bulging of the dorsal edge fragment in traumatic cranial burst fractures cases allows, in our cases, a simple and valid differentiation between osteoporotic and non-osteoporotic fractures. Further validation in a larger sample, including dual-energy X-ray absorptiometry (DXA) measurements, is necessary.

## 1. Introduction

Vertebral fractures are a relevant issue in daily practice. They can be caused by either a high-energy impact or a low-energy trauma, in combination with reduced bone quality. In multiple injured patients following a high-energy trauma, the spine is involved in up to 34% of cases [1]. In these patients, implant anchorage is easy to reach due to their good bone quality. In contrast, osteoporotic fractures are an increasing problem. In industrial countries, by the year 2050, 54% of the population will be aged 65 years or older [2]. Thus, age-related orthopedic diseases will increase accordingly. Even today, the osteoporotic vertebral fracture is the most common osteoporotic fracture. According to estimates, there are 1.7 million osteoporotic vertebral fractures each year in Europe and in the USA [3,4].

In case of cranial burst fractures (AO Type 3), posterior stabilization using pedicle screws is indicated. The biggest challenge in operative treatment is reaching a sufficient implant anchorage. Therefore, not long ago, severe osteoporotic vertebral fractures, even with neurologic manifestation (radiculopathy and claudication), were considered inoperable by many spine surgeons [5].

Recently new techniques and devices have been introduced. The most common method to enhance pedicle screw anchorage is screw augmentation. Polymethylmethacrylate-based (PMMA) bone cement is used to increase the bone-screw interface and thus increase the stability of the entire instrumentation. Several biomechanical studies showed a higher static (pull-out force) and dynamic (cycles to failure) strength [2,5,6]. Additionally, different clinical studies pointed out a reduced frequency in the risks of implant loosening and a significant reduction of the correction loss [7]. Nevertheless, pedicle screw augmentation is connected to relevant risks and complications (e.g., prolonged operation time with increased blood loss and risk of infection, cement leakage, cement embolism) [8]. Therefore, it is necessary to identify the patients that might best benefit from this procedure.

The diagnostic algorithm after spinal injuries includes plain X-rays and computer tomography (CT). The CT scan can give a first impression on the local bone quality, but to get objective values a quantitative computer tomography (qCT) or a dual-energy X-ray absorptiometry (DXA) are necessary.

In clinical routine, the authors had the impression that the morphology of the posterior edge looked different in traumatic osteoporotic fractures compared to non-osteoporotic fractures. The posterior edge of the cranial fragment showed a “bulging” in the osteoporotic cases, whereas non-osteoporotic had sharp contours. Therefore, we hypothesized that a differentiation between osteoporotic and non-osteoporotic AO Type A3 vertebral fractures is possible by using a simple morphological analysis.

## 2. Material and Methods

This study was approved by the responsible ethics committee (Ethics Committee of the Medical Association of Westphalia-Lippe and the Westphalian Wilhelms University in Münster—2019-582-f-S). According to paragraph 6 of the Health Data Protection Act NRW patient consent was waived by the Ethics Committee of the Medical Association of Westphalia-Lippe and the Westphalian Wilhelms University in Münster. We declare, that all methods were carried out in accordance with relevant guidelines and regulations (e.g., Health Data Protection Act).

The analysis of the CT data was carried out by two independent and blinded examiners. One examiner analyzed the Hounsfield units and the other analyzed the morphology of the posterior edge fragment edge.

### 2.1. CT-Datasets

For this study, patients with an AO type A3 fracture were retrospectively identified by searching the medical database since 2010 using the ICD 10 and OPS codes. This search revealed 86 patients with cranial burst fractures of the thoraco-lumbar spine (from the 10th thoracic to the 2nd lumbar vertebra) and with computer tomography scans including sagittal reconstruction as DICOM datasets available. The CT scanner used was a Siemens Somatom (Siemens Health Care GmbH, Erlangen, Germany), scans were performed using the clinical routine settings for computer tomography of the spine or whole-body computer tomography during polytrauma management. Image reconstruction was performed with kernel B60s. For further evaluation the axial and sagittal multiplanar reconstruction with a slice thickness of 2–3 mm was used.

These 86 patients (mean age 49 years, ranging from 18 to 84; 53 female, 33 male) were divided into two groups. Group I (non-osteoporotic) included 52 patients (mean age 35 years, ranging from 18 to 77; 25 female, 27 male) after high-energy trauma (e.g., car/motorcycle accident). Group II (osteoporotic) included 34 patients (mean age 70 years, ranging from 49 to 86; 28 female, 6 male) with a medical history of osteoporosis and a low-energy trauma (e.g., fall from standing height or fall on the buttocks from a low height). No DXA measurements are available of these patients, therefore Hounsfield units have been measured in the CT scans to characterize bone quality (see below).

### 2.2. Morphological Analysis—Bulging

**Figure 1:**
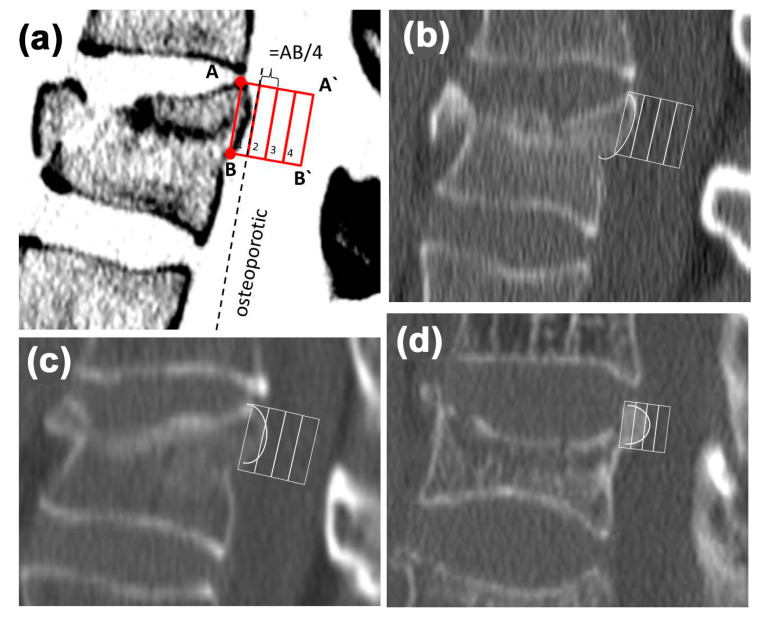
Classification of the degree of bulging. (**a**) Schematic picture showing the algorithm: defining the most cranial A and the most caudal B point of the posterior edge fragment, creating a square with the page length of the distance A to B. Subsequently, the square is divided into 4 equal boxes (1 to 4). (**b**) Sagittal reconstruction of a non-osteoporotic (box 1) traumatic vertebral fracture. (**c**,**d**) Sagittal reconstructions of osteoporotic traumatic vertebral fractures reaching box 2 and 3.

For morphologic analysis, the OsiriX software (Version 5.9, Fa. Pixmeo, Bernex, Switzerland) was used. The sagittal reconstruction (slice thickness 2 mm) was opened, the window width was set to 300 Hounsfield units (HU) and the window level to 1500 HU (bone window). The next step was to identify the correct slice. Therefore, the paramedian slice showing the most prominent shaped bulging of the posterior edge was set and controlled in the axial slice (to eliminate an accidental misplacement e.g., at the pedicel region). Afterward, the most cranial point of the posterior edge fragment (A) and the most caudal (B) were connected using a square with the page length of the distance from A to B (Figure 1). Then the bulging of the posterior edge fragment was inside the square.

To quantify the bulging, the square was divided into 4 equal parts (Figure 1). Afterward, the box into which the bulging reached was identified. If the bone touched or cut a line, it was defined as trespassing the box (Figure 1). This method evaluates the bulging in proportion to the craniocaudal extension and thus is independent from metrical parameters.

For further evaluation of the number of the box, the bulging reached value was used.

### 2.3. Radiologic Analysis—Hounsfield Units (HU)

**Figure 2:**
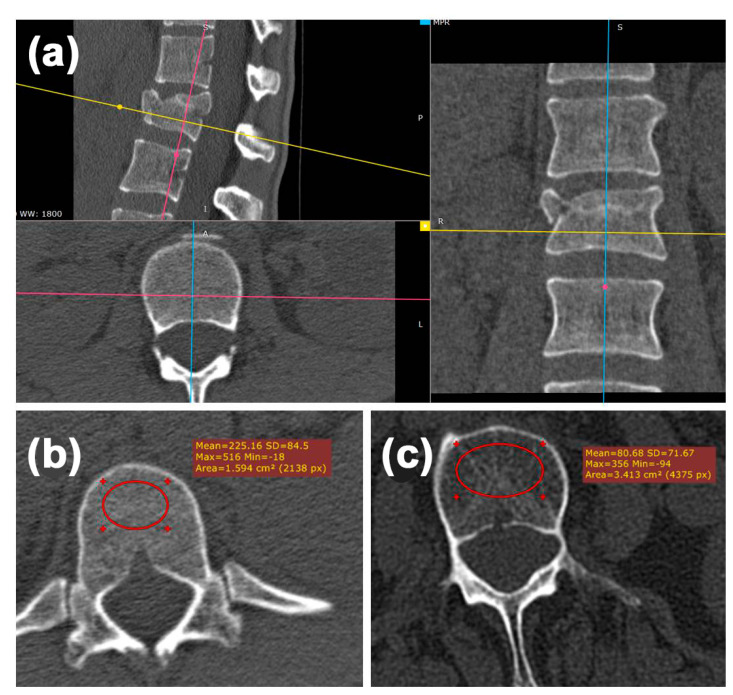
Hounsfield unit measurements in the broken vertebra, the one above and below, following the reported algorithm. (**a**) The axis have been adapted using a multiplanar reconstruction, the correct alignment was checked sagittal (blue line), transversal (yellow line) and coronal (pink line) and corrected if necessary. Subsequently, the measurements were performed in the axial slice within the defined regions of interest (red circles). (**b**) A non-osteoporotic vertebra with a mean Hounsfield unit (HU) of 225.16. (**c**) An osteoporotic vertebra with a mean HU of 80.86.

To quantify the bone density, mean Hounsfield unit (HU) of the broken vertebra (caudal the fracture), as well as the vertebral body above and below, were measured within the axial reconstruction (slice thickness 3 mm) using the OsiriX software (Version 5.9, Fa. Pixmeo, Bernex, Switzerland) (Figure 2). An elliptical region of interest (ROI) was placed in the anterior trabecular bone space using axial slices within the bone window settings (Figure 2a). The mean HU was measured over 5 slices. We avoided placing the ROI over areas of attenuation (e.g., posterior venous plexus, focal heterogeneity, fracture). The threshold to distinguish osteoporotic from non-osteoporotic fractures was chosen from the literature, with an HU < 110 characterizing an osteoporotic condition (Figure 2b,c) [9,10].

### 2.4. Statistical Methods

For the statistical analysis, Microsoft Excel (Version 14, Microsoft Cooperation, Redmond, DC, USA) and SPSS 24.0 (SPSS Inc., Chicago, IL, USA) were used. The first step was to evaluate the differences in bulging in both groups using the chi-square test. Afterward, a cut-off-value for the presence of an osteoporotic vertebral fracture was defined. For this purpose, the fourfold table was used. Sensitivity, specificity, and positive predictive value (PPV) were calculated. Regarding the HUs, after testing the normal distribution of the data (Kolmogorov–Smirnov test), the Mann–Whitney U test was carried out to identify significant differences between groups. Level of significance was set to α = 0.05.

## 3. Results

### 3.1. Patient’s Data

The mean age of the 86 patients was 49 years, ranging from 18 to 84. We enrolled in the study 53 women (mean age 52 years, range from 18 to 86) and 33 men (mean age 44 years, range from 18 to 77).

Group I (non-osteoporotic) included 52 patients with a mean age of 35 years (range from 18 to 77). In this group, there were 25 women (mean age 31 years, range from 18 to 63) and 27 men (mean age 40 years, range from 18 to 77).

Group II (osteoporotic) included 34 patients with a mean age of 70 years (range from 49 to 86). In this group, there were 28 women (mean age 72 years, range from 49 to 86) and 6 men (mean age 64 years, range from 59 to 70).

### 3.2. Morphologic Analysis—Bulging

The chi-square test showed a significant difference (*p* < 0.001) in the manifestation of the bulging between group I and II (Table 1).

The determination of a cut-off-value using the fourfold table revealed that box 2 was the most promising value (Table 2). Sensitivity was 100%, meaning that all 34 patients with osteoporosis could be identified. Specificity was 96%, as two patients without osteoporosis were false positive. Using these conditions, the PPV was 94%, meaning that in case of a positive test result, the probability of the presence of an osteoporotic fracture is 94%.

### 3.3. Radiologic Analysis—Hounsfield Units

The mean values of the Hounsfield units for all patients and both groups are shown in Table 3. The Hounsfield units of the fractured vertebrae showed significantly higher values (*p* < 0.001), especially in the osteoporotic patients (+96% versus +21% in the non-osteoporotic group). Therefore, for further evaluation, the mean value of the Hounsfield units of the vertebra above and below the fracture was used.

The chi-square test showed a significant difference (*p* = 0.002) in the Hounsfield unit measurement comparing groups I and II. Using the fourfold table (Table 2) for an HU threshold of < 110, the sensitivity was 94%, specificity was 94%, the PPV was 91%.

## 4. Discussion

Vertebral fractures, especially osteoporotic vertebral fractures, are an increasing problem in the therapy of spinal injuries. There are increasing special options for the operative treatment of osteoporotic vertebral fractures. Many of them are related to additional risks and possible complications, as well as additional costs for the health system. Therefore, it is essential to identify patients with osteoporotic fractures prior to operative therapy.

Thus, this study reports preliminary results of a simple method to identify osteoporotic vertebral fractures by analyzing the morphology of the posterior edge fragment in the sagittal reconstruction of the clinical routine computer tomography scan. In our cohort of 86 patients, we were able to show a significant difference in the bulging of the posterior edge fragment between traumatic osteoporotic and non-osteoporotic AO A3 vertebral fractures. This difference can be objectified using a simple method, which can be integrated into the clinical routine and performed by radiologists or surgeons. The method consists of drawing a square with the side length defined by the cranial and caudal point of the posterior edge fragment, which is then divided into four equal boxes, allowing the identification of osteoporotic fractures with a sensitivity of 100% and a specificity of 96% if the dorsal edge fragment bulges into the second box. Our preliminary results show a positive predictive value of 94% in our cohort. In contrast, the method of measuring the Hounsfield units in the vertebral body above and below the fracture is more sophisticated and has a sensitivity of 94%, a specificity of 94%, and a PPV of 91%.

**Figure 3:**
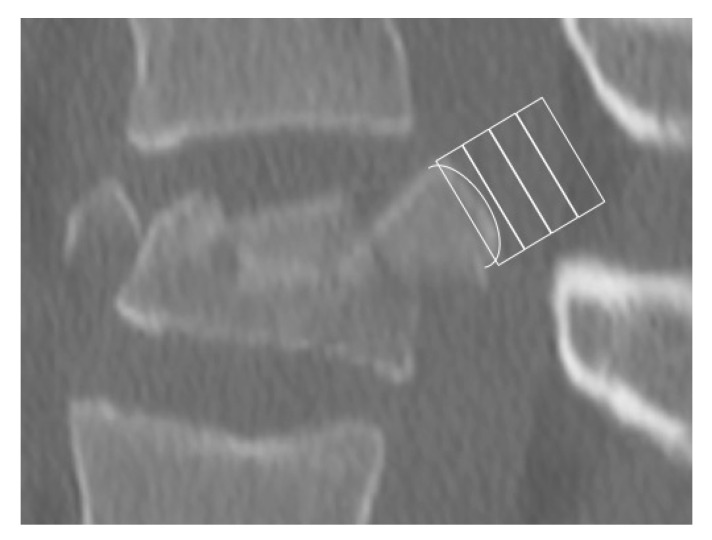
Sagittal picture of a displaced vertebral body fracture of a 17-year-old female patient after a fall from a horse. The analysis shows bulging into box 1.

One possible limitation of this method could be the dislocation of the dorsal edge fragment. In these cases, special emphasis has to be placed on the identification of the cranial and caudal point of the posterior edge fragment. Figure 3 presents a case of dislocated burst fracture of a 17-year-old female after a fall from a horse. Analysis shows bulging into box 1, which means non-osteoporotic fracture.

Traumatic osteoporotic fractures differ significantly from pathologic osteoporotic fractures. Spontaneous or pathologic osteoporotic fractures can often be treated conservatively whereas traumatic osteoporotic fractures frequently require operative stabilization. Reduced bone stock quality increases the risk of complication significantly [11]. The major issues are the screw loosening and the progressive loss of reduction, which can be addressed by using augmentation techniques and long-distance instrumentation [12]. Pedicle screw augmentation using PMMA-based bone cement is a sufficient method to enhance implant anchorage in the osteoporotic bone. Biomechanical and clinical studies showed benefits for the patient, but the connected risks and possible complications cannot be neglected. One major complication is cement leakage, which can cause a pulmonary embolism. In up to 94% of patients with augmented pedicle screws, paravertebral cement leakage can be detected by CT scan. Most of them are clinically not relevant, but in 4–8% of the cases, a pulmonary embolism was found, which has a mortality rate of up to 1.3% [13,14].

Therefore, it is necessary to have a simple to use tool for the identification of patients with a high failure risk. The gold standard to diagnose osteoporosis is the DXA method. This is an additional investigation, which is not applicable in most patients with an acute vertebral fracture. Therefore, various studies investigated the value of routine computer tomography scans in the prediction of osteoporosis. The consensus was that a routine CT scan is appropriate to distinguish osteoporotic vertebrae from non-osteoporotic. Nevertheless, the suggested cut-off values differ significantly among the studies. Schreiber et al. define a value of less than 95 Hounsfield units as an osteoporotic condition [15]. In contrast, Choi et al. defined ≤ 100 HUs as osteoporotic and Pickard et al., as well as Lee et al., defined ≤ 110 HUs as the threshold to characterize osteoporosis [9,10,16]. In a recent review article, Scheyerer et al. focus on the value of the Hounsfield units as a measure of bone density and the possible applications in spinal surgery [17]. They emphasize on the measurement in clinical routine CT scans to early diagnose and treat osteoporosis. Furthermore, in patients with vertebral fractures and HU values of <120, they suggest to use screw augmentation. Additional stabilization of ventral cages is indicated in cases with HU values <180. Nevertheless, this method also has disadvantages, there is no standardization and the values are device-specific. Furthermore, the authors recommend three measurements within each vertebra (cranial, middle, caudal) and at least the evaluation of three vertebra [17]. Thus, this measurement is time consuming and we think routine use will be difficult.

Another technique to determine the local bone quality is the intra-operative measurement of the peak torque necessary to break the local trabecular bone before inserting the pedicle screw (DensiProbe^TM^, AO Research Institute, Davos, Switzerland) [18]. The use of this technique has been investigated in different anatomical regions (hip, hindfoot, spine, humerus) in vitro. All this type of study showed a good correlation between local bone strength and radiological-determined bone quality (either from DXA or qCT). Additionally, the local bone strength negatively correlated with the implant failure load in all biomechanical studies [18,19,20,21,22,23]. For spinal instrumentation and the implantation of a dynamic hip screw, clinical application workflows to determine the local bone strength were already published [24,25]. The technique of intra-operative bone quality assessment needs to be further validated and thresholds have to be defined yet.

The limitations of this study are the small size of the groups and the biased sex distribution within the osteoporotic group. Furthermore, no information on inter- and intra-observer reliability can be given. Additionally, no DXA measurements have been performed in the osteoporotic group. Therefore, it is necessary to study a larger cohort and evaluating all patients by DXA technique to further validate our original and simple method. A prospective study is necessary to prove whether this classification can improve treatment outcomes.

## Figures and Tables

**Table 1:** Degree of bulging in both groups.

Box	Osteoporotic (*n* = 34)	Non-Osteoporotic (*n* = 51)
1	0	49 (96.1%)
2	30 (88.2%)	2 (3.9%)
3	4 (11.8%)	0
4	0	0

**Table 2:** Fourfold table for the cut-off values “box 2” and “Hounsfield unit (HU) < 110”.

Cut-Off Box 2	Osteoporosis		Total
	+	−	
Test +	34 (100%) true positive	2 (4%) false positive	36
Test −	0 (0%) false negative	50 (96%) true negative	50
Cut-Off HU < 110			
Test +	32 (94%) true positive	3 (6%) false positive	35
Test −	2 (6%) false negative	49 (94%) true negative	51

**Table 3:** Mean Hounsfield units (HU) measured in the fractured vertebra, as well as above and below (standard deviation). Measures for all patients and in both groups.

	HU Fractured Vertebra	HU Vertebra Above	HU Vertebra Below
All	184.6 (70)	147.6 (74)	137.5 (72)
Non-osteoporotic	220.9 (59)	193.9 (56)	182.9 (54)
Osteoporotic	129.1 (45)	76.8 (28)	68.2 (24)
